# National Institutes of Health Funding in Internal Medicine: Analysis of Physicians Receiving an R01 Grant Between 2008 and 2017

**DOI:** 10.7759/cureus.12842

**Published:** 2021-01-21

**Authors:** Erich J Berg, Anthony Santarelli, John Ashurst

**Affiliations:** 1 Emergency Medicine, Midwestern University, Glendale, USA; 2 Emergency Medicine, Kingman Regional Medical Center, Kingman, USA

**Keywords:** r01, grant, internal medicine, investigator, national institutes of health (nih)

## Abstract

Introduction

As the world’s largest funding source for biomedical research, the National Institutes of Health (NIH) supports physician-scientists with a discipline-specific R01 grant. Recently, scholarly activity disparities regarding investigator degree and gender have been highlighted in the medical literature among allopathic and osteopathic investigators of various medical backgrounds. We aimed to assess trends in internal medicine NIH R01 grants over the past decade.

Methodology

Internal medicine R01 funding was retrospectively obtained from a centralized online NIH database encompassing 2008 through 2017. Principal investigators (PIs) were then categorized by gender and academic degree(s). Two-way analysis of variance was used to analyze NIH grant funding trends over the time period studied.

Results

A total of 5,089 NIH R01s were awarded to internal medicine PIs, with an average value per grant of $469,270. Awardees were predominantly male (71.5%, 3,639/5,089). Most awards were issued to PIs with an MD degree (62.4%, 3,173/5,089), followed by PhD degree (36.3%, 1,845/5,089). DOs accounted for five awards over the time period studied (0.15%). MDs were awarded higher funding than PhDs ($466,494 and $421,576, p < 0.001), and females were awarded higher amounts than males ($462,771 and $444,868, p < 0.001). Investigators who held a second degree received more funding than PIs with a single degree ($476,693 and $439,693, p < 0.001).

Conclusion

In the decade under investigation, both gender and degree disparities existed within NIH R01 funding for PIs in the field of internal medicine, and osteopathic representation accounted for a paucity of R01 funding.

## Introduction

Over the last several years, a disparity in the amount of scholarly activity produced by allopathic and osteopathic physicians has emerged. Recent data have shown that very few osteopathic physicians serve as either the first or senior author in published original research manuscripts in emergency medicine, obstetrics and gynecology, pediatrics, and neurosurgery [1-4]. Additionally, no osteopathic physicians have been awarded a National Institutes of Health (NIH) R01 grant in emergency medicine, family medicine, general surgery, or obstetrics and gynecology in the previous decade [5-8]. Although we do not anticipate substantial differences in grants awarded to internal medicine investigators, no data currently exist on the scholarly activity between osteopathic and allopathic physicians within the specialty of internal medicine. Thus, the primary study objective was to determine the number and percentage of osteopathic and allopathic physicians who were awarded an NIH R01 grant in internal medicine from 2008 through 2017. We hypothesize that principal investigators (PIs) with MD and/or PhD degrees would be awarded more R01 grants per year than osteopathic physicians. Secondarily, gender and degree demographics of NIH R01 grant awardees were reviewed.

## Materials and methods

Following Institutional Review Board approval, the NIH Research Portfolio Online Reporting Tools Expenditures and Results (RePORTER) search engine (available from: projectreporter.nih.gov) was queried using “internal medicine” as a keyword. Specifically, new R01 grants between the fiscal years 2008 and 2017 were obtained, and the corresponding PIs were categorized based upon gender (male or female) and terminal degree (PhD, DO [osteopathic physician], MD [allopathic physician], or other [DMD, DVM]). MD/PhD awardees were considered MDs when compared to PhD-only investigators. The public profile from the affiliated institution of each PI was reviewed to ensure gender and degree accuracy. The total grant amount (USD) awarded to each PI was recorded; PIs were not excluded if they had been awarded more than one grant over the timeframe studied. Prior to statistical analysis, 122 awards were removed: 39 as outliers (z > 3.0) for dollar value, 71 for possessing a primary degree other than MD or PhD, and 12 for unspecified sex. All analyses were conducted on the remaining 4,967 awards.

Comparisons of funding amounts were computed using a 2 × 2 repeated-measures analysis of variance. All analyses were conducted using the SPSS software version 27 (IBM, Armonk, New York, USA). All dollar values are reported as an average of the groups with one standard deviation. The z-score was calculated as Z = (x - M)/SD, where X is the observed value, M is the sample mean, and SD is the sample standard deviation. Normality was assessed by evaluation of the histograms and Q-Q plots.

## Results

Over the decade studied, a total of 5,089 NIH R01 grants were found to be awarded to internal medicine investigators with an average value of $469,270. The awardees were predominantly male 71.5% (3,639/5,089), while 28.3% were female (1,438/5,089) and 0.2% (12/5,089) could not have their sex confirmed. When assessed for terminal degree, the majority of awards were issued to MDs (62.4%, 3,173/5,089) and PhDs (36.3%, 1,845/5,089). DOs accounted for five awards over the time period studied (0.15%). A total of 901 (17.7%) grants were awarded to investigators with combined MD/PhD degrees.

The average funding amount from 2008 to 2017 showed a linear increase (p < 0.001, Table 1) uniformly for sex (male, female) and advanced degrees (MD, PhD). MDs ($466,494 + $182,751) were consistently awarded higher funding than PhDs ($421,576 + $143,626, p < 0.001). Females ($462,771 + $175,731) were consistently awarded higher funding than males ($444,868 + $168,497, p < 0.001). Investigators who held a second degree ($476,693 + $187,481) were consistently awarded more than those holding a single degree ($439,693 + $162,760, p < 0.001). This effect is non-uniform between MD and PhD primary degree holders (p < 0.001), where holding a second degree leads to a greater increase in funding for PhDs (Δ$72,296, p < 0.001) than it does for MDs (Δ$15,482, p = 0.021).

Of the osteopathic physicians who were awarded an R01 in internal medicine, three (60%) were female, three held a master’s degree, and three graduated from the Chicago College of Osteopathic Medicine. Each (5/5) osteopathic physician had completed a fellowship in an internal medicine subspecialty. As a group, osteopathic physician-researchers were awarded a total of $2,621,587, with each PI receiving, on average, $524,317 to fund their individual studies. The mean number of published manuscripts indexed in PubMed in the 10 years prior to receiving the first R01 numbered 43, and each author had been in practice for 20 years prior to receiving their first award.

**Table 1:** Number of NIH R01 funding awards tallied from NIH-REPORTER broken down by primary degree type and sex. Data are reported as N (average award value + standard deviation).

	PhD		MD	
	Female	Male	Female	Male
Total	665 ($439,096 + $158,225)	1,166 ($411,584 + $133,638)	741 ($484,017 + $187,651)	2,395 ($461,073 + $180,904)
2008	66 ($390,419 + $139,221)	113 ($372,232 + $111,721)	64 ($453,248 + $204,386)	255 ($412,327 + $159,591)
2009	78 ($433,775 + $156,844)	128 ($411,002 + $123,400)	89 ($471,259 + $183,266)	319 ($441,899 + $194,221)
2010	60 ($376,219 + $133,706)	119 ($382,070 + $117,853)	56 ($492,516 + $215,615)	250 ($453,768 + $185,283)
2011	50 ($435,542 + $168,887)	80 ($397,382 + $120,233)	62 ($445,456 + $147,280)	212 ($466,647 + $173,869)
2012	60 ($404,631 + $116,004)	102 ($392,941 + $147,984)	60 ($471,074 + $177,707)	203 ($465,743 + $194,411)
2013	54 ($461,313 + $153,338)	113 ($412,806 + $121,129)	59 ($465,743 + $161,546)	194 ($448,892 + $170,682)
2014	62 ($447,877 + $177,445)	97 ($407,900 + $134,985)	77 ($529,544 + $186,387)	224 ($473,540 + $185,531)
2015	69 ($473,568 + $143,711)	112 ($426,964 + $142,536)	77 ($499,449 + $230,855)	233 ($469,764 + $153,210)
2016	76 ($468,452 + $166,752)	152 ($439,757 + $146,093)	104 ($493,179 + $164,087)	270 ($492,131 + $179,884)
2017	90 ($475,678 + $177,347)	150 ($446,826 + $140,608)	93 ($497,225 + $190,699)	235 ($492,576 + $191,148)

## Discussion

The medical specialty with the highest number of positions offered to first-year (PGY-1) residents is internal medicine; this program collectively offered 8,697 PGY-1 seats to MD and DO seniors in the 2020 National Resident Matching Program match. Of all specialties, internal medicine garners the greatest number of MD applicants and the second-most applications by DOs applying to any medical specialty [9].

As a comparatively large field, internal medicine encompasses multiple subspecialties in which investigators conduct a diverse array of sponsored investigations. However, with regard to osteopathic representation in scholarly avenues, the results of this study are similar to prior observations of R01 grant awards in the disciplines of emergency medicine, family medicine, general surgery, and obstetrics and gynecology. Despite an abundance of interest from osteopathic medical student applicants toward the field of internal medicine, osteopathic physicians were under-represented in R01 research awards during the decade studied.

Historically, scholarly activity has not manifested in the osteopathic community at the institutional level. From 2006 to 2010, 28 osteopathic medical schools produced a mere 1,843 published manuscripts, an average of approximately 13 annual publications per school [10]. Among more than a dozen types of various educational institutions, osteopathic medical schools ranked last in NIH research funding in 2011. Possibly, compared to allopathic schools, a comparably low number of NIH grants are awarded to graduates of osteopathic medical schools because the research infrastructure at such institutions is lacking or absent. While the five osteopathic awardees in this report had considerable prior research and clinical experience, bibliometrics for allopathic physicians were not inspected. This may prove a promising research question for additional reports investigating the comparison in prior research output for physician-scientists who receive R01 grants.

The absence of R01 grants among DO physician-scientists reflects a considerable problem facing the osteopathic community today. The uninspiring pursuit of research activity among DOs may be due to a myriad of factors, including a lack of research support and funding, access to mentors, or success in previous scholarly endeavors. Migrative thinking will be required to adapt to the ACGME merger, where DO applicants now compete directly with their allopathic counterparts for shared residency positions. Challenges involved in bolstering osteopathic research include infrastructure and support, incentives, and expectations placed upon students and faculty. Scientific inquiry within the osteopathic profession should be encouraged beyond the bounds of osteopathic manipulative medicine itself, and venture to areas on par with primary care colleagues from the fields of emergency medicine, family practice, obstetrics and gynecology, and pediatrics to maximize scholarly output.

Awardees were primarily male, but females were awarded higher dollar amounts per grant. This gender disparity in scholarly activity within internal medicine may simply reflect the overall representation of a larger male percentage in the specialty [9]. It may also reflect a greater number of male applications, which was unavailable for analysis. The presence of a second degree constituted an increase in award funding for both MD and PhD PIs with a greater increase for PhDs, but causes cannot be ascertained by the scope of this paper and further discussion is warranted. Possibly, a greater amount of education through fellowship or more robust research experience are modulators of NIH R01 funding, but cannot be definitively determined through this analysis.

Limitations

The authors can only comment on the decade studied; osteopathic physicians may have received an NIH R01 subsequent to the timeframe included in this analysis. Additionally, institutional displays of an investigator’s biographical information may have been outdated, leading to errors related to the quality of data abstraction. Further, the present study does not include the number of osteopathic physicians who may have applied for an NIH R01 grant and were not selected recipients based upon merit. Finally, the mechanisms at play that have produced such sex differences within grant funding were not investigated but are an intriguing avenue for future research.

## Conclusions

While award amounts increased for MDs and PhDs in internal medicine over the decade studied, very few recipients of an NIH R01 grant held a degree in osteopathic medicine. The majority of osteopathic physicians who were awarded an NIH R01 grant held an advanced degree, completed a fellowship, and each had numerous publications prior to their initial award. While gender disparities were observed in the number of awardees favoring male investigators and grant amounts favoring female investigators, future research should be aimed at determining the publication trends within internal medicine and its subspecialties to better understand the possible degree disparities which exist within the field.
